# CV-EEGNet: A Compact Complex-Valued Convolutional Network for End-to-End EEG-Based Emotion Recognition

**DOI:** 10.3390/s26030807

**Published:** 2026-01-26

**Authors:** Wenhao Wang, Dongxia Yang, Yong Yang, Yuanlun Xie, Xiu Liu, Yue Yu, Kaibo Shi

**Affiliations:** 1 School of Electronic Information and Electrical Engineering, Chengdu University, Chengdu 610106, China; wangwenhao@cdu.edu.cn (W.W.); yangdongxia@cdu.edu.cn (D.Y.); yuyue@cdu.edu.cn (Y.Y.); shikaibo@cdu.edu.cn (K.S.); 2 College of Computer Science, Chengdu University, Chengdu 610106, China; 212023081202002@cdu.edu.cn; 3 School of Mathematics, Southwest Jiaotong University, Chengdu 611756, China

**Keywords:** electroencephalogram, emotion recognition, complex-valued neural network, raw signal

## Abstract

In electroencephalogram (EEG)-based emotion recognition tasks, existing end-to-end approaches predominantly rely on real-valued neural networks, which mainly operate in the time–amplitude domain. However, EEG signals are a type of wave, intrinsically including frequency, phase, and amplitude characteristics. Real-valued architectures may struggle to capture amplitude–phase coupling and spectral structures that are crucial for emotion decoding. To the best of our knowledge, this work is the first to introduce complex-valued neural networks for EEG-based emotion recognition, upon which we design a new end-to-end architecture named Complex-valued EEGNet (CV-EEGNet). Beginning with raw EEG signals, CV-EEGNet transforms them into complex-valued spectra via the Fast Fourier Transform, then sequentially applies complex-valued spectral, spatial, and depthwise-separable convolution modules to extract frequency structures, spatial topologies, and high-level semantic representations while preserving amplitude–phase relationships. Finally, a complex-valued, fully connected classifier generates complex logits, and the final emotion predictions are derived from their magnitudes. Experiments on the SEED (three-class) and SEED-IV (four-class) datasets validate the effectiveness of the proposed method, with t-SNE visualizations further confirming the discriminability of the learned representations. These results show the potential of complex-valued neural networks for raw-signal EEG emotion recognition.

## 1. Introduction

Electroencephalogram (EEG) directly reflects the temporal dynamics of brain activity, offering high temporal resolution and strong physiological relevance. Therefore, EEG-based emotion recognition represents a significant research direction in affective computing, demonstrating broad application potential in scenarios such as intelligent human–computer interaction, mental health monitoring, and personalized services [1,2,3].

Existing EEG-based emotion recognition methods can be primarily divided into two categories. The first type of method, as described in references [4,5,6], is based on artificial features. These methods utilize features like differential entropy (DE) or power spectral density (PSD) as model inputs and have achieved high accuracy on multiple benchmark datasets [7,8]. However, artificial features rely heavily on prior assumptions during the extraction process, often eliminating a large amount of potentially useful information [9,10,11]; In addition, they are highly sensitive to data distribution, have limited generalization, and often experience significant performance degradation under dataset switching, task switching, or different session conditions. Such methods struggle to meet the stability and robustness requirements demanded by real-world applications.

The second type is an end-to-end method based on raw EEG, which can avoid the above problems and has important research significance that should not be ignored [12,13]. This type of method preserves the complete information of the original signal and utilizes the powerful nonlinear modeling ability of deep networks to automatically learn feature representations. However, compared to the first type, this type of method has not been widely explored in the field of EEG emotion recognition. We speculate that the reason may be that the relevant indicators of the end-to-end method are not as good as the carefully selected artificial features for offline datasets, leading to insufficient confidence among researchers in this method. In other fields such as motor imagery, sleep staging, and depression detection [14,15,16], end-to-end EEG decoders such as EEGNet [17], ShallowConvNet [18], and CTNet [19] have achieved satisfactory performance, demonstrating the important research value of raw data modeling.

**Figure 1:**
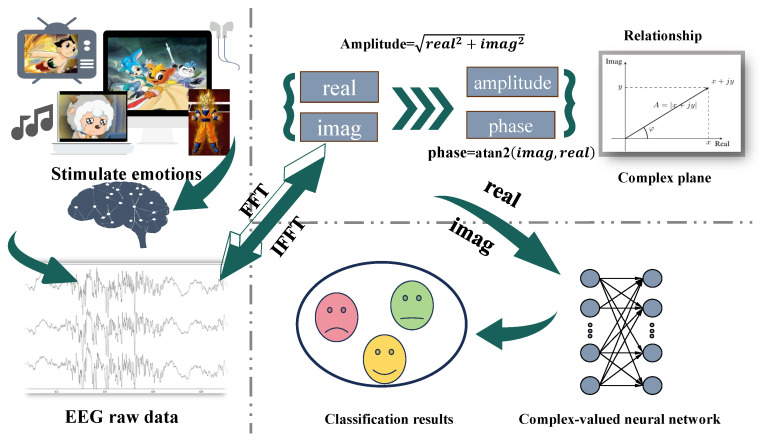
Pipeline of EEG emotion recognition. Emotional stimuli evoke EEG responses, which are transformed into complex-valued frequency representations through Fourier analysis. These complex-domain representations, characterized by amplitude and phase information, are subsequently processed by a complex-valued neural network to achieve end-to-end emotion recognition.

However, in EEG-based emotion recognition tasks, the above raw-signal decoding methods did not achieve satisfactory results. This may be attributed to the complexity of the experimental paradigms, the greater number of emotion categories, and the subtler signal variations involved in emotion recognition tasks. As shown in Figure 1, EEG signals are waveforms characterized by inherently low signal-to-noise ratio [20]. When transformed into the frequency domain, they are represented by complex-valued spectra whose real and imaginary parts jointly determine the amplitude and phase at each frequency, forming essential descriptors of neural oscillatory activity. These spectral characteristics play a central role in EEG-based emotion analysis. However, conventional real-valued neural networks (RVNNs)—such as EEGNet—operate only on time-domain samples and amplitudes, limiting their ability to capture such oscillatory representations, especially under small-sample conditions. Even when researchers manually convert EEG into frequency and phase spectra, RVNNs still process these inputs as independent real-valued features, without preserving the intrinsic amplitude–phase coupling inherent in the complex domain. This mismatch results in the loss of physically meaningful spectral relationships and restricts the model’s ability to exploit emotion-relevant neural dynamics.

For this problem, complex-valued neural networks (CVNNs) may offer a viable solution. CVNNs are a natural extension of RVNNs, whose inputs, parameters, and operations are defined in the complex domain [21]. Compared with real-valued networks that treat frequency-domain features as independent real-valued inputs, complex-valued neural networks (CVNNs) jointly learn the real and imaginary components of a signal, thereby preserving the intrinsic amplitude–phase relationships encoded in the complex domain [22]. Such structural consistency provides a natural advantage when modeling signals characterized by frequency-domain structure, phase dependencies, or inherently complex representations. In fields such as computer vision and signal processing [23,24,25], CVNN-based methods have been explored and demonstrated certain advantages.

It can be intuitively seen that CVNN is compatible with EEG signals. Similar to the idea of fuzzy systems characterizing different patterns through multiple rules [26,27], we may be able to learn the multi-component structure of EEG signals from the perspective of complex-valued domain modeling. Therefore, based on the concept of complex domain, we extend the classical EEGNet architecture to complex-valued form and propose a new end-to-end EEG emotion recognition model—CV-EEGNet. This method directly starts from the raw EEG signal and obtains the complex frequency domain representation of the signal through Fast Fourier Transform, as shown in Figure 1. Then, complex-valued spectral convolution and complex-valued spatial convolution are then employed to extract frequency features and spatial topological relationships from the EEG signal, respectively. Afterwards, the complex-valued depthwise-separable convolution module is used to further extract deeper semantic information. Finally, emotion classification is completed through complex-valued fully connected layers and modulus normalization. This method not only maintains the advantages of end-to-end modeling but also fully utilizes the expressive power of complex-valued networks for phase amplitude coupling structures. According to the literature review, this marks the first exploratory application of CVNNs in EEG-based emotion recognition, providing a promising and effective approach for this field.

The main contributions of this paper can be summarized as follows:

1. To the best of our knowledge, we introduce complex-valued neural networks into EEG-based emotion recognition for the first time, proposing CV-EEGNet, an end-to-end model that jointly models frequency–phase–amplitude relationships for more robust emotion decoding.
2. We design complex-valued spectral, spatial, and depthwise-separable convolution modules that collaboratively extract discriminative frequency, spatial, and semantic information.
3. Extensive experiments on the SEED [28] and SEED-IV [29] datasets verify the effectiveness of complex-valued neural networks for raw EEG-based emotion recognition, providing the empirical evidence that complex-domain modeling is a viable and promising paradigm for this task.

## 2. Related Works

### 2.1. RVNNs for EEG-Based Emotion Recognition

Based on real-valued neural networks, deep learning has been widely applied in various scenarios such as medical image [30], facial expression recognition [31], multimodal learning [6,32], and robust learning with noisy [33] or weak supervision [34]. It has also promoted the rapid development of EEG emotion recognition field. Many researchers have proposed corresponding models based on the temporal and spatial characteristics of EEG signals.

In temporal modeling, recurrent structures such as LSTM and GRU are widely adopted to extract long-range dependencies in emotional EEG signals. ATDD-LSTM introduces an attention mechanism to focus on emotionally relevant channels and employs domain discrimination to alleviate inter-subject and inter-session discrepancies [35]. In spatial modeling, graph convolutional networks (GCNs) have been used to represent the non-Euclidean relationships between EEG electrodes. DGCNN learns a dynamic adjacency matrix to capture functional connectivity among channels [36]. SparseDGCNN further imposes a sparsity constraint to emphasize local connectivity [37], while RGNN incorporates biologically inspired graph structures and regularization strategies to enhance cross-subject generalization [38]. Several works also attempt to integrate both spatial and temporal information. EWT-3D–CNN–BiLSTM–GRU–AT constructs 3D EEG representations using empirical wavelet transform and applies CNN, BiLSTM, GRU, and attention modules to jointly learn spatiotemporal features [39]. ERDL combines multi-layer GCNs and LSTM to model both graph-domain structures and temporal dynamics [40].

Although these real-valued models have advanced EEG-based emotion recognition, they fundamentally operate in the amplitude domain and cannot effectively capture the phase, frequency, or their coupling properties inherent in EEG signals. This limits their representational capacity for complex emotional brain patterns.

### 2.2. CVNNs for Other Fields

CVNNs have demonstrated superior performance in modeling structured signals by jointly learning real and imaginary components. Leveraging magnitude, phase, and frequency-domain features, CVNNs have been widely applied across communication, radar, vision, acoustics, and industrial systems [41].

In communication, SlimCVNN achieves high-accuracy emitter identification with a significantly reduced model size [42], while a hybrid CVNN-RF framework introduces an early-exit mechanism to balance accuracy and efficiency for RF fingerprinting [43]. In imaging tasks, a CV-CNN has been developed for PolSAR classification, effectively exploiting the complex structure of polarimetric SAR data [44]. From a theoretical standpoint, single-layer CVNNs have been shown to construct nonlinear decision boundaries with faster convergence and stronger expressive power compared to real-valued counterparts [45]. Moreover, a CVAE combined with sequential Monte Carlo enables distribution-aware multistep prediction and uncertainty quantification in industrial soft sensor applications [46].

These studies collectively establish the effectiveness of CVNNs in handling non-stationary, frequency-rich, and phase-sensitive signals. These advantages suggest that CVNNs may also be promising for EEG analysis, which inherently contains complex-valued characteristics after Fourier or Hilbert transformation.

### 2.3. CVNNs for EEG Analysis

Complex-valued neural networks (CVNNs) have attracted increasing attention in EEG analysis due to their ability to jointly model amplitude and phase information in the complex domain. In steady-state visual evoked potential (SSVEP) tasks, Ikeda and Washizawa proposed the CVCNN model, which performs complex-valued convolution directly in the frequency domain [47]. This approach alleviates the dependence on predefined frequency templates in CCA or power-spectrum-based methods and achieves superior classification performance under multiple stimulation frequencies. In sleep stage classification, Zhang et al. introduced the unsupervised complex-valued convolutional neural network (CUCNN), which learns multi-band rhythmic structures without requiring large amounts of manually labeled data, demonstrating strong performance in multi-stage sleep recognition [48]. In addition, Taran et al. employed the optimized flexible analytic wavelet transform (OFAWT) to extract complex-domain features aligned with physiological rhythms, further improving sleep staging accuracy [49]. In the clinical EEG domain, CVNN-based methods also show promising results. Du et al. proposed a hybrid CVNN framework that integrates DFT-based complex representations with real-valued convolutional blocks, enabling more effective modeling of epileptic-related frequency variations while reducing the overall model size [50]. Similarly, Peker et al. utilized dual-tree complex wavelet features combined with complex-valued classifiers to build an automated epilepsy detection system, achieving high accuracy, sensitivity, and specificity [51].

Overall, existing studies demonstrate that CVNNs offer clear advantages in EEG tasks characterized by well-defined frequency structures, such as SSVEP recognition, sleep staging, and epilepsy detection. However, the application of CVNNs in EEG emotion recognition is still limited or even may not exist. We have searched on the Internet or in the relevant literature but did not find any, as if it had been forgotten by everyone.

### 2.4. Phase-Related EEG Emotion Recognition

Some studies have shown that emotional processes are closely related to oscillatory phase dynamics and frequency-specific interactions in EEG signals. Phase synchronization measures such as phase locking value (PLV) and phase lag index (PLI) have been widely used to construct functional connectivity patterns, which are further modeled using convolutional or graph-based neural networks to capture spatial relationships among EEG channels [52,53,54]. In addition, cross-frequency coupling mechanisms, particularly phase–amplitude coupling (PAC), have been investigated to characterize interactions between low-frequency phase and high-frequency amplitude components, providing discriminative statistical features for emotion recognition [55]. More recently, STRFLNet incorporate PLI-based functional connectivity as a structural prior and combine dynamic-static graph modeling with temporal transformers to jointly learn spatial and temporal dependencies [56].

Despite their effectiveness, existing phase-aware and frequency-domain approaches primarily rely on handcrafted phase descriptors or predefined coupling indices, and the subsequent neural networks operate exclusively in the real-valued domain. As a result, amplitude and phase information are not jointly optimized within a unified end-to-end learning framework, motivating the adoption of complex-valued neural networks for EEG emotion recognition.

## 3. Method

**Figure 2:**
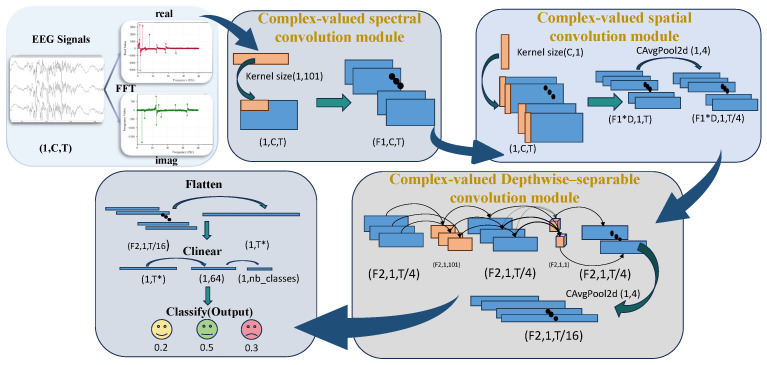
Overview of the proposed CV-EEGNet. Raw EEG signals are converted into complex-domain representations using FFT and sequentially processed through the following: (1) a complex-valued spectral convolution module, (2) a complex-valued spatial convolution module, and (3) a complex-valued depthwise-separable convolution module. The final features are flattened and classified through a complex-valued fully connected layer to obtain emotion predictions.

In this work, we introduce the CVNN method to reconstruct the classical EEG decoding network–EEGNet [17], and a corresponding network named CV-EEGNet is proposed for EEG emotion recognition. As shown in Figure 2, the model employs complex-valued network layers as the core feature extractor and classifier, enabling joint learning of the real and imaginary components of EEG signals while preserving the intrinsic amplitude–phase consistency encoded in the complex domain. This end-to-end method shows stronger EEG signal decoding ability, verifying that CVNN is a possible direction in EEG emotion recognition. In the following content, we first introduce several layer structures within complex-valued neural networks, followed by a detailed explanation of our method.

### 3.1. Complex-Valued Neural Networks

In this subsection, a complex-valued convolution layer (CConv), a complex-valued average pooling layer (CAvgPool), and a complex-valued fully connected layer (CLinear) are introduced. These components are collaboratively integrated to establish a comprehensive complex-valued learning framework through which end-to-end feature extraction and emotion classification are achieved.

#### 3.1.1. Complex-Valued Convolution Layer

The CConv operates under the condition that both the convolution kernels and input features are complex-valued, enabling simultaneous encoding of amplitude variations and phase differences within a single operation. Let the input feature be H∈C:

(1) $$H=H_{r}+iH_{i}$$

where $H_{r}$ and $H_{i}$ denote the real and imaginary parts of the input features, and i is the imaginary unit. The convolution kernel W∈C is defined as

(2) $$W=W_{r}+iW_{i}$$

Similarly, $W_{r}$ and $W_{i}$ represent the real and imaginary parts of the convolution kernel, respectively. Thus, the output feature Y∈C of the complex-valued convolution can be expressed as

(3) $$\begin{array}{cc} Y & =CConv\left(H\right)=H*W=\left(H_{r}*W_{r}-H_{i}*W_{i}\right)+i\left(H_{r}*W_{i}+H_{i}*W_{r}\right) \end{array}$$

where the ∗ denotes the convolution operation.

#### 3.1.2. Complex-Valued Pooling Layer

The CAvgPool layer is used to perform downsampling in the complex domain, simultaneously considering both the real and imaginary parts. This operation reduces dimensionality while preserving the intrinsic amplitude–phase correspondence of complex features. The operation of CAvgPool can be expressed as follows:

(4) $$Y=\mathrm{CAvgPool}\left(H\right)=\frac{1}{N}\sum_{n=1}^{N}H_{r,n}+i\frac{1}{N}\sum_{n=1}^{N}H_{i,n}$$

where *N* denotes the number of elements within the pooling window, and $H_{r,n}$ is the *n*-th element in $H_{r}$.

#### 3.1.3. Complex-Valued Fully Connected Layer

The CLinear layer extends real-valued linear mapping to the complex domain, enabling the joint modeling of real and imaginary signal parts. By preserving both amplitude and phase relations, it captures richer representational dynamics and improves robustness for frequency-dependent neural signals such as EEG. Similarly to the convolution operation, if the bias term is defined as $b=b_{r}+ib_{i}$, the complex-valued linear transformation can be expressed as

(5) $$\begin{array}{cc} Y & =\mathrm{CLinear}\left(H\right)=WH+b=\left(W_{r}H_{r}-W_{i}H_{i}+b_{r}\right)+i\left(W_{r}H_{i}+W_{i}H_{r}+b_{i}\right) \end{array}$$

### 3.2. Complex-Valued EEGNet

EEGNet [17] is a well-known early network for processing EEG signals that has had a significant impact in the field of motor imagery, but its performance in EEG emotion recognition has been suboptimal. In this paper, we introduce CVNNs to enhance it and propose CV-EEGNet. This network simultaneously learns the amplitude–phase distribution patterns across different frequency components.

Given an EEG dataset $D={\left\{\left(X_{i},y_{i}\right)\right\}}_{i=1}^{N}$, each sample $X_{i}\in R^{C\times T}$ represents a multi-channel time-domain EEG segment with *C* channels and *T* time points. The $y_{i}$ is its corresponding label. Let $x_{c}\left(t\right)$ denote the signal of the *c*-th channel at time *t*. The frequency-domain representation of each channel is obtained by applying the Fast Fourier Transform (FFT):

(6) $${\tilde{x}}_{c}\left(f\right)=\sum_{t=0}^{T-1}x_{c}\left(t\right)\;e^{-j\frac{2\pi ft}{T}},\;f=0,1,\ldots ,T-1,$$

(7) $${\tilde{x}}_{c,f}={\mathrm{real}}_{c,f}+j\;{\mathrm{imag}}_{c,f},$$

Each Fourier coefficient ${\tilde{x}}_{c}\left(f\right)\in C$ is a complex number. For clarity, we decompose it into its real and imaginary components. Thus, Equation (7) is simply the Cartesian-form decomposition of the FFT coefficient defined in Equation (6). By stacking the spectra of all channels, we obtain the complete complex-domain representation:

(8) $${\tilde{X}}_{i}={\left[{\tilde{x}}_{1},\;{\tilde{x}}_{2},\;\ldots ,\;{\tilde{x}}_{C}\right]}^{⊤}\in C^{C\times F},$$

where F=T denotes the number of frequency bins. Each complex element ${\tilde{x}}_{c,f}$ contains both amplitude and phase information:

(9) $$A_{c,f}=\left|{\tilde{x}}_{c,f}\right|=\sqrt{{\mathrm{real}}_{c,f}^{2}+{\mathrm{imag}}_{c,f}^{2}},$$

(10) $$\Phi_{c,f}=\arg\left({\tilde{x}}_{c,f}\right)=atan2\;\left({\mathrm{imag}}_{c,f},\;{\mathrm{real}}_{c,f}\right).$$

where $A_{c,f}$ and $\Phi_{c,f}$ represent amplitude and phase, respectively. Notably, the second dimension of ${\tilde{X}}_{i}$ corresponds to frequency rather than time.

As shown in Figure 2, we feed the complex-valued data ${\tilde{X}}_{i}$ into the CV-EEGNet. Firstly, after the real-valued data undergoes FFT, the first convolution of EEGNet is no longer to extract temporal features, but to extract phase and amplitude features at different frequencies, which is more appropriately called the complex-valued spectral convolution module. Next, the complex-valued spatial convolution module is used to capture spatial relationships between electrode channels. The complex-valued depthwise-separable convolution module is then used to extract deeper features. Finally, the complex-valued fully connected layer integrates high-level features, and we converts the network output into a real probability distribution through modular operations.

#### 3.2.1. Complex-Valued Spectral Convolution Module

In EEGNet, a temporal convolution module with a kernel of [1, length] is used to extract temporal features from EEG signals in the horizontal dimension. After the EEG signal undergoes Fourier transform, it has been converted into the complex form of $\tilde{X}\in C^{C\times F}$. So, in CV-EEGNet, the complex-valued spectral convolution module with a kernel of [1, length] extracts spectral information. Specifically, the complex form of EEG signals is first input into CConv (Kernel size: [1, length]) to extract frequency-domain features. Subsequently, a complex-valued batch normalization (CBN) layer performs joint affine transformations on the real and imaginary components. Then, a complex-valued activation function (CReLU) is used to introduce nonlinear mapping to enhance the feature representation ability of the model. The overall computation process of this module can be defined as

(11) $$y_{1}=\mathrm{CReLU}\left(\mathrm{CBN}(\mathrm{CConv}(X))\right)$$

The activation function is defined as

(12) $$\mathrm{CReLU}\left(H\right)=\mathrm{ReLU}\left(H_{r}\right)+i\cdot \mathrm{ReLU}\left(H_{i}\right)$$

#### 3.2.2. Complex-Valued Spatial Convolution Module

This module uses a complex-valued convolutional layer with a kernel size of [C, 1], where C is the number of electrode channels of the EEG signals. Therefore, in the process of convolution, the convolutional layer only pays attention to the relationships between channels, without involving information in the frequency dimension. In fact, the EEG signals recorded by electrodes in different brain regions exhibit significant synchronicity and spatial dependence [57,58]. Therefore, this module is crucial for modeling the potential relationships between channels. Specifically, the module performs complex-valued convolution operations along spatial dimensions, capturing joint response patterns across electrode channels. Subsequently, the CBN is also applied to jointly standardize the real and imaginary parts, enhancing numerical stability. A CReLU introduces nonlinear transformations to improve feature expressiveness. Finally, a CAvgPool performs downsampling to further compress features. The formulation of this module can be expressed as follows:

(13) $$y_{2}=\mathrm{CAvgPool}\left(\mathrm{CReLU}\left(\mathrm{CBN}(\mathrm{CConv}\left(y_{1}\right))\right)\right)$$

#### 3.2.3. Complex-Valued Depthwise-Separable Convolution Module

In order to reduce the number of parameters and obtain a compact model, EEGNet uses a depthwise-separable convolution module. Correspondingly, we also adopt the same structure, which is beneficial for demonstrating the potential of CVNNs in the field of EEG emotion recognition. So, a complex-valued depthwise–separable convolution module is used as the deeper feature extraction module. This structure comprises two components: complex-valued depthwise convolution (Depthwise CConv) and complex-valued pointwise convolution (Pointwise CConv), which are used to extract local spectral features and inter-channel interaction relationships within the feature map, respectively.

Specifically, this module first feeds the previously extracted features into two complex-valued convolutions with kernel sizes [1, length’] and [1, 1] to further extract deeper features. Subsequently, CBN and CReLU are used for nonlinear mapping and normalization of deep features. Finally, the feature map is further compressed through CAvgPool as input to the classifier. These module can be simply formulated as

(14) $$\begin{array}{c} z=\left\{\underset{\mathrm{point}-\mathrm{wise}}{\underbrace{{\mathrm{CConv}}^{\mathrm{point}}\left(\underset{\mathrm{depth}-\mathrm{wise}}{\underbrace{{\mathrm{CConv}}^{\mathrm{depth}}\left(y_{2}\right)}}\right)}}\right\},\\ \;y_{3}=\mathrm{CAvgPool}\left(\mathrm{CReLU}\left(\mathrm{CBN}(z)\right)\right) \end{array}$$

#### 3.2.4. Complex-Valued Fully Connected Classifier

In the classification module, after the preceding three stages of convolution, the extracted complex-valued features are first flattened and then fed into a two-layer complex-valued fully connected network. The first fully connected layer produces a compact representation of high-dimensional complex-domain features, while the second outputs the final complex-valued prediction. The computation process is formulated as follows:

(15) $$y_{4}=\mathrm{CLinear}\left(\mathrm{CLinear}(\mathrm{Flatten}\left(y_{3}\right))\right)$$

To ensure compatibility with real-valued loss functions, the network output undergoes a modulus operation, as defined below, which extracts the magnitude of the complex-valued output, yielding a real-valued representation that can be further normalized into a probability distribution. This transformation yields the final real-valued prediction probabilities used for emotion classification.

(16) y^=|y4|=(Re(y4))2+(Im(y4))2

where Re(y4) and Im(y4) are the real and imaginary parts of $y_{4}$, respectively.

#### 3.2.5. Loss Function

The cross-entropy loss function is employed as the primary optimization objective of the model. Let *M* denote the number of training samples. The standard cross-entropy loss is defined as follows:

(17) $$L=-\frac{1}{M}\sum_{i=1}^{M}\sum_{k=1}^{K}y_{i,k}\log{\hat{y}}_{i,k}$$

where *K* is the number of classes, $y_{i}$ denotes the one-hot ground-truth label of the *i*-th sample, and $\hat{y}$ represents the predicted probability distribution over all classes.

## 4. Experiment

### 4.1. Datasets and Protocol

This study is conducted on two public emotion EEG datasets, SEED [28] and SEED-IV [29], released by the Brain-Like Computing and Machine Intelligence (BCMI) Lab at Shanghai Jiao Tong University. The SEED dataset consists of EEG recordings from 15 subjects (7 males and 8 females), each participating in three independent sessions conducted at different times. During the experiments, participants were asked to watch validated emotional film clips designed to elicit three affective states: positive, neutral, and negative. Each session includes 15 trials, with 5 trials per emotion. The SEED-IV dataset also contains EEG data from 15 subjects, but the emotional categories are extended to four states: neutral, sad, fearful, and happy. Each subject participated in three sessions, and each session includes 24 trials. Similarly to SEED, the emotional stimuli were induced by film clips under identical acquisition settings. In both datasets, the EEG signals were recorded from 62 channels following the international 10–20 system, at a sampling rate of 1000 Hz, and subsequently downsampled to 200 Hz.

In the experimental design, a subject-dependent protocol is employed, where a separate model is trained and tested for each subject. For the SEED dataset with 15 trials of one subject, the first 9 trials are used for training and the remaining 6 trials for testing. For the SEED-IV dataset with 24 trails, with each session including 24 trials, we design the partition strategy by selecting the last 2 trials of each emotion as the test set and using the remaining trials for training. This strategy maintains balanced distributions among different emotion categories. We used the raw signals with a duration of 1 s as the sample and employed sliding windows with an overlap of 0.5 s for segmentation. Then, all samples are normalized using z-score normalization.

### 4.2. Implementation Details

For hyperparameter of CV-EEGNet, we have detailed the hyperparameters in the model in Table 1. Here, we introduce hyperparameter setting for training. A joint optimization of batch size and learning rate was performed to achieve the best performance across different datasets and sessions. Specifically, the batch size was selected from {16, 32, 64, 128}, and the learning rate was searched within the range of [0.001, 0.035]. Considering the balance between training stability, convergence speed, and validation performance, a batch size of 64 was finally adopted as a unified setting. The model was trained for 120 epochs using GPU parallelization to accelerate optimization. All experiments were conducted on a workstation running Windows 10, equipped with an NVIDIA GeForce RTX 4080 SUPER GPU (16 GB VRAM) and a 13th-generation Intel Core i7 processor (32 cores, 3.2 GHz). The software environment was configured with Python 3.11.13 and PyTorch 2.5.1, using CUDA 12.1 and cuDNN 9.0.1. All models were trained using the Adam optimizer with default hyperparameters unless otherwise specified.

### 4.3. Performance of Proposed Method

To comprehensively verify the effectiveness of the proposed model, we conducted experiments on two EEG emotion datasets, SEED and SEED-IV, and presented the experimental results. We used line charts to display our model’s accuracy on each subject’s data and the results across different sessions. Furthermore, we synthesized the model’s predictions for all subjects’ samples to construct the confusion matrix, hoping to obtain certain findings from it.

#### 4.3.1. Detail Results on SEED

**Figure 3:**
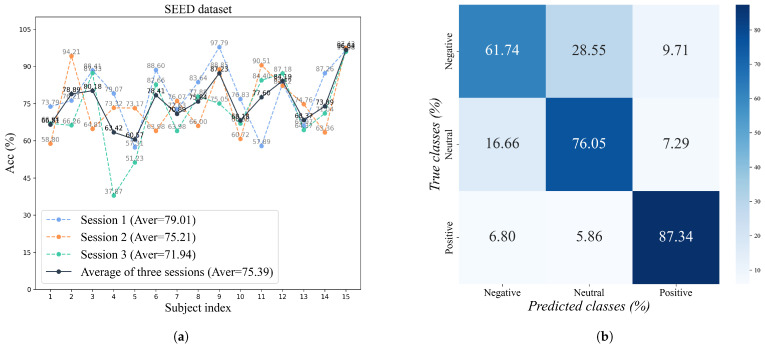
Experimental results on the SEED dataset. (**a**) Accuracy (%) of each subject across three sessions. (**b**) Confusion matrix (%) for the samples of all subjects.

The detailed experimental results of the proposed method on the SEED dataset are illustrated in Figure 3. Figure 3a presents the classification accuracies of 15 subjects over three recording sessions. The proposed model achieved average accuracies of 79.01%, 75.21%, and 71.94% in Session 1, Session 2, and Session 3, respectively, with an overall mean of 75.39%. Firstly, from the perspective of the subjects, it can be observed that there are significant differences in the model among different subjects. For example, the accuracy of three sessions for subject 15 is high at over 90%, whereas Subject 4’s Session 3 accuracy was only 37.87%. This indicates significant individual differences in EEG emotion recognition, and our experimental results conform to general patterns. Furthermore, overall trends were consistent, with most participants maintaining a performance of over 70% in all three sessions, indicating that the model has good generalization performance and cross session stability.

Figure 3b presents the confusion matrix results for the three emotion categories on session 1 of the SEED dataset. We gathered data from all subjects in the session to form this composite confusion matrix. From the figure, it can be seen that the model has the most accurate recognition in the ‘Positive’ category, with an accuracy rate of 87.34%, suggesting it effectively captures the EEG feature distribution associated with negative emotions. The ‘Neutral’ category follows with an accuracy of 76.05%, while the ‘Negative’ category achieves 61.74% accuracy. It is worth noting that there is a certain degree of confusion (28.55%) between negative and neutral emotions, which may be due to the small differences in their characteristics in EEG signals. Overall, the model has a certain ability to distinguish between the three emotion categories, especially in the recognition of positive emotions.

#### 4.3.2. Detailed Results on SEED-IV

**Figure 4:**
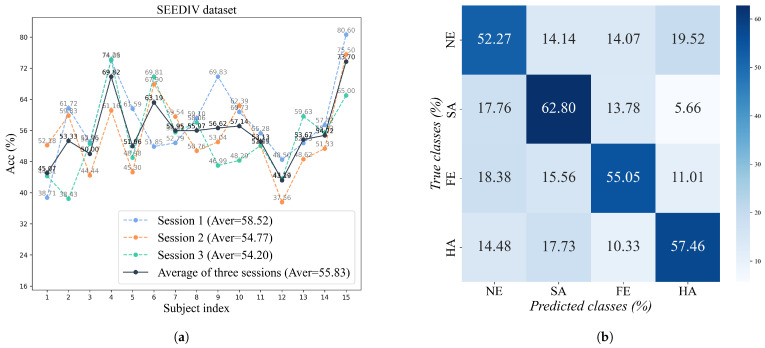
Experimental results on the SEED-IV dataset. (**a**) Accuracy (%) of each subject across three sessions. (**b**) Confusion matrix (%) for the samples of all subjects.

Figure 4a shows the classification accuracy performance of the proposed model on the SEED-IV dataset with 15 participants and three different sessions. Overall, the average accuracies for the three sessions were 58.52%, 54.77%, and 54.20%, respectively, with a composite average of 55.83%. Compared to the SEED dataset, the accuracy on this dataset is generally lower. This is primarily because the SEED-IV dataset expands the emotion categories from three to four, increasing task difficulty. Additionally, the boundaries between emotion categories become more ambiguous, especially with numerous transitional states between similar emotions. There are still significant individual differences among the subjects, with subjects such as S4, S6, and S15 having significantly higher accuracy than others, while subjects such as S1 and S12 have lower recognition performance. These indicate that, although the model has certain stability in complex-valued emotion recognition tasks, it is still affected by individual differences in EEG responses, and there is still a lot of room for improvement in the model.

Figure 4b is the confusion matrix for the CV-EEGNet’s four-classification task on Session 1 of the SEED-IV dataset, also combining samples from all subjects. It can be observed that the recognition performance for the “Sad” emotion category is the best, with an accuracy of 62.80%, while the recognition performance for “Neutral” is the lowest at 52.27%. Additionally, the “Fear” and “Happy” categories achieved intermediate levels of 55.05% and 57.46%, respectively. This confusion matrix reveals frequent misclassifications: “Neutral” is often confused with “Sad” (14.14%), “Fear” (14.07%), and “Happy” (19.52%). Similarly, “Fear” is frequently misclassified as ‘Neutral’ (18.38%) and “Sad” (15.56%). This suggests that certain emotions share overlapping or similar neural patterns in EEG signals, posing challenges for the model in distinguishing between borderline emotions. Overall, the model achieves fundamentally effective differentiation among the four emotion categories. However, there remains room for improvement when confronting tasks with increased emotion categories and more complex sample distributions, particularly in reducing cross-classification rates.

### 4.4. Comparison with Other Methods

#### 4.4.1. Performance Comparison on SEED and SEED-IV Datasets

As shown in Table 2, we compared CV-EEGNet with other methods on the SEED and SEED-IV datasets. The comparison methods include traditional machine learning approaches (SVM, Random Forest), graph neural network-based methods (DGCNN [36], SparseDGCNN [37], RGNN [38]), and convolutional neural network models (EEGNet [17], ShallowConvNet [18], CTNet [19], FBSTCNet [59]).

The results indicate that traditional SVM and Random Forest performed relatively poorly on both datasets, particularly on the SEED-IV dataset where accuracy for all three classes fell below 20%. This suggests their inability to effectively model the temporal characteristics and inter-channel relationships inherent in raw EEG signals. Graph neural network methods (DGCNN, SparseDGCNN, and RGNN) outperformed traditional methods on the SEED dataset. However, due to their reliance on handcrafted features like differential entropy in their original design, their recognition capabilities are limited when directly processing raw signals. This is particularly evident on the SEED-IV dataset, where their accuracy generally falls below 45%.

Convolutional neural network methods demonstrated more stable overall performance across both datasets. Our proposed method achieved 79.01%, 75.21%, and 71.94% accuracy across the three experiments on the SEED dataset, with an average accuracy of 75.39%. On the SEED-IV dataset, the accuracy rates for the three sessions were 58.52%, 54.77%, and 54.20%, respectively, with an average of 55.83%. Specifically, FBSTCNet achieved comparable accuracy to Our approach, but their respective *p*-values are greater than 0.05, indicating no statistically significant superiority between them. In addition, as shown in Table 3, the model complexity and inference time of FBSTCNet far exceed our method. The comprehensive results indicate that our approach can achieve good accuracy while preserving an appropriate number of parameters and a relatively fast computation speed.

#### 4.4.2. Comparison of Model Complexity

For a more comprehensive comparison, we also evaluated the model complexity and inference efficiency of each method, with results shown in Table 3. Comparison metrics include the number of parameters (Params), computational load (FLOPs), and single inference time (Inf. Time). We simultaneously report the average recognition accuracy on both the SEED and SEED-IV datasets to facilitate a holistic comparison of the models’ strengths and weaknesses.

The results demonstrate that while traditional GNNs relying on manually extracted features offer significant advantages in terms of parameter count and floating-point operations, their accuracy remains insufficient for processing raw data. Furthermore, although CNN-based methods like CTNet and FBSTCNet exhibit certain accuracy advantages, their parameter counts and FLOPs are significantly higher than other approaches. For example, CTNet possesses over 156 K parameters and 871 M FLOPs, while FBSTCNet’s FLOPs also exceed 260 M, increasing computational burden during deployment. In contrast, our proposed method maintains high classification performance with a lighter-weight architecture, containing only approximately 16 K parameters and 31 M FLOPs. This achieves a favorable accuracy–complexity trade-off among the compared deep learning approaches.

#### 4.4.3. Comparison on t-SNE

**Figure 5:**
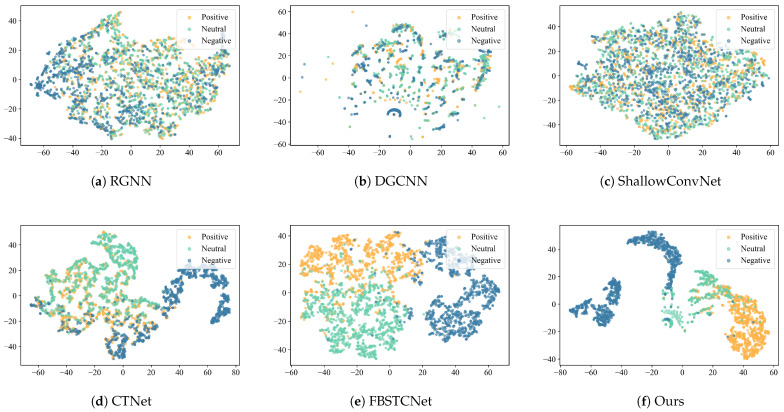
t-SNE visualization of learned EEG representations from different models on the SEED dataset. Each color corresponds to one emotion category (Positive, Neutral, Negative). Our model shows clearer separation and more compact clusters.

To intuitively illustrate the feature separability learned by different models, we visualize the latent representations of three emotion categories on the SEED dataset using t-SNE, as shown in Figure 5. Each point represents one sample, and different colors indicate different emotion labels. The latent features are extracted from the last feature layer before the classifier of each model. To ensure a fair comparison, all models are visualized using the same t-SNE configuration and identical experimental settings, with the perplexity set to 30 and the maximum number of iterations set to 1000. A fixed random seed (random_state = 42) is used to guarantee reproducibility, and no additional dimensionality reduction or post-processing is applied. We present the visualization results of session 2 for the 11th subject, which is randomly selected for illustration.

On the whole, the sample distributions of RGNN, DGCNN, and ShallowConvNet are relatively dispersed, with blurred boundaries between emotion categories and significant overlapping regions among samples. This indicates that the models extract limited discriminative features when processing raw EEG signals. While CTNet exhibits a clearer clustering structure, some emotion categories remain mixed, particularly between “Positive” and the other two emotion categories. FBSTCNet further improves the feature clustering structure, showing some differentiation among the three emotion categories, though local overlaps persist. In contrast, our model exhibits a clearer clustering structure in the feature space, with the three emotion categories distinctly distributed overall. Samples within each category are compact, and boundaries between categories are well-defined. This result indicates that our model can more effectively extract discriminative emotional features when modeling EEG, thereby enhancing classification accuracy.

### 4.5. Discussion

#### 4.5.1. The Ablation of Complex Value

To further validate the effectiveness of complex-valued neural networks in EEG-based emotion recognition, we conducted a comprehensive comparison between the baseline EEGNet (real-valued) and the proposed CV-EEGNet on both the SEED and SEED-IV datasets, each containing three sessions. As shown in Table 4, the average recognition accuracy of each subject (S1–S15) and the corresponding gain (Δ) are reported. The results demonstrate that CV-EEGNet consistently outperforms EEGNet across both datasets. On the SEED dataset, the proposed model achieves significant average accuracy improvements of approximately 23.23% over EEGNet with complex-value calculation. On the SEED-IV dataset, which involves a more challenging four-class classification task, CVNN-EEGNet still exhibits notable superiority, with an average improvement of about 10.21%. The gains observed in subjects such as S3, S6, and S10 further confirm that complex-valued representations effectively capture the intrinsic amplitude–phase relationships of EEG signals, thereby enhancing the model’s ability to generalize across emotional states and recording sessions. These results collectively verify that the incorporation of complex-valued operations substantially improves the model’s capacity for discriminative and stable emotion representation learning.

#### 4.5.2. The Ablation of Model’s Structure

To verify the importance of proposed complex-valued modules in emotion recognition tasks, we conducted ablation experiments on the three core components of CV-EEGNet. The results are shown in Table 5. When any module is removed, the performance of the model on SEED and SEED-IV datasets decreases, which verifies the synergy of three types of complex-valued modules and the necessity of their application in end-to-end complex domain modeling. Among them, the missing complex-valued spatial convolution module has a significant impact on performance, indicating that in the complex domain, spatial structure plays a key role in emotion discrimination. Secondly, removing the depthwise-separable convolution module will also weaken its feature expression ability, indicating that this module plays an important role in further integrating high-level complex-valued semantic features.

One point is worth noting here. As shown in experimental setup 2 of Table 5, after removing the spatial convolution module, the model’s accuracy dropped significantly. Merely setting 2 may not ultimately prove the importance of spatial convolutions, as removing them would result in feature maps not being compressed, resulting in a sharp increase in the number of neurons within the CLlinear layer in the final classifier. At this point, the total number of model parameters is 3.17 M, which is much higher. We suspected that the model’s performance might be adversely affected by the sharp increase in the number of neurons within the CLinear layer. Therefore, we designed an additional experiment: replacing the spatial convolution module with average pooling to prevent excessive neuron growth in the classifier and return the model parameter count to a normal state (Params 51.74 K). The experimental results refuted our suspicion, confirming that the significant performance drop indeed occurred after removing the module.

The ablation experiments here actually have a slight limitation. Since each module can compress feature maps, removing any single module results in the classifier having more parameters than the full model, as shown in Table 5. Relying solely on the table cannot fully explain the problem. To further demonstrate, we conducted experiments using a single module, as shown in Table 6. The results showed that although the spatial convolution module compressed the largest feature dimension, it achieved the best classification results. These results in Table 5 and Table 6 comprehensively confirm the crucial role of the complex-valued spatial convolution module in raw EEG emotion recognition.

It is worth noting that this does not mean that other modules are not important. On the SEED dataset, when comparing setting 4 in Table 5 and setting 2 in Table 6, adding a complex spectral convolution module separately to the spatial convolution module can improve the accuracy of SEED by 0.68%. Furthermore, when comparing setting 4 and setting 2 in Table 5, adding a complex depth separable module can further improve accuracy by 2.07%.

#### 4.5.3. Analysis of Different Input Representations

We compared the performance of different input representations and network structures on the SEED dataset in Table 7 to validate the effectiveness of CV-EEGNet. From the perspective of input representation (Settings 2/3/4), compared to directly using raw time-domain EEG signals, mapping EEG signals to complex-domain representations can achieve higher classification accuracy, whether by using only real or imaginary parts. This indicates that complex-domain representations help to more clearly characterize discriminative features related to emotional states. At the network architecture level, by comparing the results of “Raw data → EEGNet” and “Raw data → CV-EEGNet”, the latter has a higher accuracy of 13.39% than the former, and the corresponding *p*-value is less than 0.01. It can be found that even with consistent inputs, complex-valued neural networks can still significantly improve model performance, indicating that CV-EEGNet is better to traditional real-valued networks in feature modeling and representation capabilities. Further integrating the experimental results from both input representation and network structure perspective, it can be seen that when complex domain EEG signals are used as inputs and combined with complex-valued neural networks for end-to-end modeling, the model achieves optimal performance. This result indicates that there is a good synergistic effect between complex input representation and a complex-valued network structure, which can more fully explore the emotion related information contained in EEG signals, thus verifying the effectiveness and application potential of complex-valued neural networks in EEG emotion recognition tasks.

#### 4.5.4. The Limitations

Although CV-EEGNet has achieved competitive performance in EEG emotion recognition tasks, there are still some limitations that deserve further research. Firstly, the model relies on FFT to map the original EEG signal to the complex frequency domain for modeling. This process is based on the assumption of global stationarity, and the characterization of transient or strong non-stationary features that may exist during emotional induction is still limited. In contrast, analytical signals constructed based on Hilbert transform can form complex-valued representations in the time domain, which is expected to better characterize the instantaneous dynamic characteristics of signals. In the future, its combination with complex-valued neural networks can be further explored. Secondly, compared to real-valued networks with the same structure, complex-valued neural networks still have certain computational complexity and engineering implementation costs, which may pose challenges for practical deployment. Finally, this study only demonstrated on empirical evidence that EEG emotions recognition in the complex domain are effective. The underlying mechanisms and whether they inherently outperform real-valued neural networks are not yet clear, necessitating further investigation.

## 5. Conclusions

This paper proposes CV-EEGNet, a complex-valued end-to-end framework designed to overcome the limitations of traditional real-valued networks by preserving the complex-domain structure of EEG signals and the intrinsic amplitude–phase relationships. The model maps raw EEG signals to the complex spectral domain via FFT and constructs key modules including complex-valued spectral convolution, complex-valued spatial convolution, and complex-valued depthwise-separable convolution to collaboratively extract frequency-domain structure, cross-channel spatial topology, and high-level complex semantic information. Extensive experimental results demonstrate that CV-EEGNet achieves competitive performance on both SEED and SEED-IV datasets while maintaining low parameter size and computational overhead, highlighting the exceptional expressive power of complex neural networks for EEG emotion recognition tasks. Ablation studies further validate the necessity of each complex module, among which the complex-valued spatial convolution module plays a more important role.

This work indicates that complex-valued neural networks may represent a potential research direction for end-to-end EEG emotion recognition, but further validation through additional studies is still required. In future research, we plan to construct more strong complex-valued network architectures, incorporate attention mechanisms, implement contrastive learning, and integrate them with information bottleneck theory to further advance the exploration of complex-valued deep learning in the fields of affective computing and neural signal decoding.

## Figures and Tables

**Table 1:** Architecture of the CV-EEGNet model.

Block	Layer	Input	Filter/Kernel	Output Shape	Activation
Spectral Conv	cConv2d	(1,C,T)	(1,101)	$\left(F_{1},C,T\right)$	CReLU
	cBatchNorm2d	$\left(F_{1},C,T\right)$	–	$\left(F_{1},C,T\right)$	–
Spatial Conv	cConv2d (Depthwise)	$\left(F_{1},C,T\right)$	(C,1)	$\left(D\times F_{1},1,T\right)$	CReLU
	cBatchNorm2d	$\left(D\times F_{1},1,T\right)$	–	$\left(D\times F_{1},1,T\right)$	–
	cAvgPool2d	$\left(D\times F_{1},1,T\right)$	(1,4)	$\left(D\times F_{1},1,T/4\right)$	–
Depthwise-Separable Conv	cConv2d (Depthwise)	$\left(D\times F_{1},1,T/4\right)$	(1,101)	$\left(D\times F_{1},1,T/4\right)$	–
	cConv2d (Pointwise)	$\left(D\times F_{1},1,T/4\right)$	1×1	$\left(F_{2},1,T/4\right)$	CReLU
	cBatchNorm2d	$\left(F_{2},1,T/4\right)$	–	$\left(F_{2},1,T/4\right)$	–
	cAvgPool2d	$\left(F_{2},1,T/4\right)$	(1,4)	$\left(F_{2},1,T/16\right)$	–
Classifier	Flatten	$\left(F_{2},1,T/16\right)$	–	(192)	–
	CLinear	192→64	–	(64)	CReLU
	CLinear	64→3	–	(3)	–

C=62 denotes the number of EEG channels; T=200 is the temporal length of the input sequence; $F_{1}=8$ is the number of spectral convolution filters; $F_{2}=16$ is the number of separable convolution filters.

**Table 2:** Classification accuracy (Acc/Std, %) of different models on the SEED and SEED-IV datasets across three sessions.

Model	SEED (Acc/Std, %)				SEED-IV (Acc/Std, %)			
	Session 1	Session 2	Session 3	Average	Session 1	Session 2	Session 3	Average
SVM	46.27/08.02	45.03/07.51	45.34/06.78	45.55 */07.25	18.37/02.41	11.31/03.62	13.50/03.42	14.39 */03.15
Random Forest	45.16/09.74	44.72/09.21	45.91/09.14	45.26 */09.36	20.17/03.02	11.12/03.92	15.53/06.47	15.91 */04.47
DGCNN [36]	46.80/07.10	44.53/06.89	43.80/07.35	45.04 */07.11	39.79/05.53	36.62/04.77	34.31/04.32	36.90 */04.87
SparseDGCNN [37]	48.91/09.52	46.42/09.01	47.32/08.94	47.55 */09.16	36.70/05.51	37.44/07.93	36.28/07.65	36.81 */07.03
RGNN [38]	54.81/10.40	51.06/10.82	53.43/11.02	53.10 */10.75	43.49/06.40	39.38/09.74	37.04/06.74	39.97 */07.63
EEGNet [17]	53.72/14.20	51.01/14.96	51.75/15.72	52.16 */14.96	50.26/12.77	44.18/10.77	42.43/11.53	45.62 */11.69
ShallowConvNet [18]	72.53/14.51	53.34/19.59	53.26/20.58	59.71 */18.23	51.41/13.90	37.87/08.98	36.98/06.23	42.09 */09.70
CTNet [19]	75.69/13.63	66.68/11.70	63.89/17.82	68.75 */14.38	49.39/09.59	41.38/08.20	42.70/10.74	44.49 */09.51
FBSTCNet [59]	78.59/11.57	**75.30/11.80**	**72.35/15.45**	**75.41/12.94**	55.74/11.43	52.67/12.66	**55.70/12.59**	54.70/12.23
Ours	**79.01/11.87**	75.21/12.31	71.94/14.50	75.39/12.89	**58.52/10.09**	**54.77/09.40**	54.20/09.63	**55.83/09.71**

Bolded numbers represent the highest accuracy rate, while underlined numbers indicate the second-highest accuracy rate. “*” represents the significantly differences in mean value compared to ours (*p* < 0.01, paired *t*-test). On SEED-IV, the *p*-value of FBSTCNet relative to us is 0.066981, while on SEED, our *p*-value relative to FBSTCNet is 0.487953.

**Table 3:** Comparison of model complexity and classification performance on the SEED and SEED-IV datasets.

Model	Params (K)	FLOPs (M)	Inf. Time (s)	SEED (Acc/Std)	SEED-IV (Acc/Std)
DGCNN [36]	6.43	10.22	0.0011	45.04/07.11	36.90/04.87
RGNN [38]	28.07	1.59	0.0081	53.10/10.75	39.97/07.63
SparseDGCNN [37]	8.14	373.95	0.0012	47.55/09.16	36.81/07.03
ShallowConvNet [18]	101.20	28.39	0.0002	59.71/18.23	42.09/09.07
CTNet [19]	156.24	871.48	0.0028	68.75/14.38	44.49/09.51
FBSTCNet [59]	32.87	268.77	0.0056	**75.41/12.94**	54.70/12.23
Ours	16.38	31.36	0.0013	75.39/12.89	**55.83/09.71**

Acc and Std denote the average accuracy (%) and standard deviation across subjects, respectively. Bolded numbers represent the highest accuracy rate, while underlined numbers indicate the second-highest accuracy rate.

**Table 4:** Comparison of average classification accuracy (%) with and without using complex values on the SEED and SEED-IV datasets. Each subject’s accuracy is averaged over three sessions.

Subject	EEGNet (SEED)	CV-EEGNet (SEED)	Δ	EEGNet (SEED-IV)	CV-EEGNet (SEED-IV)	Δ
S1	39.51	66.51	+27.00	40.71	45.07	+4.36
S2	67.43	78.89	+11.46	50.41	53.32	+2.91
S3	61.89	80.18	+18.19	31.35	50.00	+18.65
S4	49.31	63.42	+14.11	60.60	69.82	+9.22
S5	48.03	60.57	+12.54	44.14	51.96	+7.82
S6	49.81	78.41	+18.60	49.76	63.19	+13.43
S7	48.03	70.89	+22.86	49.30	55.95	+6.65
S8	51.19	75.84	+24.63	42.46	55.97	+13.51
S9	54.97	87.23	+32.26	52.19	56.62	+3.43
S10	42.88	68.18	+25.30	32.48	57.14	+24.66
S11	51.63	77.60	+25.97	46.28	53.13	+6.85
S12	47.19	84.19	+37.00	35.72	43.19	+7.47
S13	44.71	68.37	+23.66	37.01	53.67	+16.66
S14	56.07	73.88	+17.81	42.03	54.72	+12.69
S15	69.88	96.65	+26.77	69.88	73.70	+3.82
Mean	52.16	75.39	+23.23	45.62	55.83	+10.21

Δ denotes the absolute accuracy gain (%) of CV-EEGNet over EEGNet averaged across three sessions.

**Table 5:** Average accuracy (%) and standard deviation of ablation experiments on SEED and SEED-IV datasets.

	Spectral	Spatial	Depthwise-Separable	SEED	SEED-IV	Params/FLOPs
				ACC/Std (%)	ACC/Std (%)	
1	✗	✓	✓	74.89/13.18	54.43/12.39	15.54 K/2.71 M
2	✓	✗	✓	56.79/16.24	39.56/09.14	3.17 M/12.13 M
3	✓	(mean)	✓	55.96/12.14	39.37/07.28	51.74 K/3.94 M
4	✓	✓	✗	73.50/12.94	52.36/10.50	53.35 K/31.11 M
5	✓	✓	✓	75.39/12.89	55.83/09.71	16.38 K/31.36 M

A cross (✗) indicates that the model does not contain the corresponding module, while a check mark (✓) indicates that it does. “(mean)” denotes replacing spatial convolution with average pooling in the spatial dimension for dimensionality reduction.

**Table 6:** The results of using single module separately on the SEED and SEED-IV datasets.

	Spectral	Spatial	Depthwise-Separable	SEED	SEED-IV	Params/FLOPs
				ACC/Std (%)	ACC/Std (%)	
1	✓			47.42/11.59	37.43/06.32	793.96 K/4.60 M
2		✓		72.82/14.61	51.58/11.93	52.52 K/3.18 M
3			✓	54.92/11.78	35.48/08.12	3.48 M/73.61 M

A check mark (✓) indicates that the model only contain the corresponding module for recognition. This experiment demonstrates the ability of the single module in EEG emotion recognition.

**Table 7:** Average accuracy (%) of different representations of inputs on the SEED dataset.

Setting	Input Representation	ACC/Std (%)
1	Raw data → CV-EEGNet	65.55/16.15
2	EEG_Real (FFT real part) → EEGNet	67.67/15.16
3	EEG_Imag (FFT imaginary part) → EEGNet	70.90/14.88
4	Raw data → EEGNet	52.16/14.96
5	EEG_Real & Imag → CV-EEGNet	75.39/12.89

The results are reported as mean accuracy and standard deviation across three sessions.

## Data Availability

The data for this study were obtained from publicly available datasets. The SEED dataset is available at https://bcmi.sjtu.edu.cn/home/seed/seed.html (accessed on 8 May 2015), and the SEED-IV dataset is available at https://bcmi.sjtu.edu.cn/home/seed/seed-iv.html (accessed on 8 February 2018).

## Abbreviations

The following abbreviations are used in this manuscript:

EEG	Electroencephalogram
CV-EEGNet	Complex-Valued EEGNet
DE	Differential Entropy
PSD	Power Spectral Density
RVNNs	Real-Valued Neural Networks
CVNNs	Complex-Valued Neural Networks
GCNs	Graph Convolutional Networks
SSVEP	Steady-State Visual Evoked Potential
SVM	Support Vector Machine
BiLSTM	Bidirectional Long Short-Term Memory Network
LSTM	Long Short-Term Memory
CV-CNN	Complex-Valued Convolutional Neural Network
OFAWT	Optimized Flexible Analytic Wavelet Transform
CConv	Complex-Valued Convolution Layer
CAvgPool	Complex-Valued Average Pooling Layer
CLinear	Complex-Valued Fully Connected Layer
CBN	Complex-Valued Batch Normalization
CReLU	Complex Activation Function
Depthwise CConv	Complex-Valued Depthwise Convolution
Pointwise CConv	Complex-Valued Pointwise Convolution
BCMI	Brain-Like Computing and Machine Intelligence
Params	Parameters
FLOPs	Computational Load
Inf. Time	Single Inference Time
